# Chronic Binge Alcohol Administration Dysregulates Hippocampal Genes Involved in Immunity and Neurogenesis in Simian Immunodeficiency Virus-Infected Macaques

**DOI:** 10.3390/biom6040043

**Published:** 2016-11-09

**Authors:** John K. Maxi, Matt Dean, Jovanny Zabaleta, Krzysztof Reiss, Gregory J. Bagby, Steve Nelson, Peter J. Winsauer, Francesca Peruzzi, Patricia E. Molina

**Affiliations:** 1 Department of Physiology, Louisiana State University Health Sciences Center, New Orleans, LA 70112, USA; jmaxi@lsuhsc.edu (J.K.M.); gbagby@lsuhsc.edu (G.J.B.); 2 Department of Genetics, Louisiana State University Health Sciences Center, New Orleans, LA 70112, USA; mdean3@lsuhsc.edu; 3 Department of Pediatrics and Stanley S. Scott Cancer Center, Louisiana State University Health Sciences Center, New Orleans, LA 70112, USA; jzabal@lsuhsc.edu (J.Z.) kreiss@lsuhsc.edu (K.R.); 4 Comprehensive Alcohol Research Center, Louisiana State University Health Sciences Center, New Orleans, LA 70112, USA; snelso1@lsuhsc.edu (S.N.); pwinsa@lsuhsc.edu (P.J.W.); 5 Department of Pharmacology and Experimental Therapeutics, Louisiana State University Health Sciences Center, New Orleans, LA 70112, USA; 6 Department of Internal Medicine, Louisiana State University Health Sciences Center, New Orleans, LA 70112, USA; fperuz@lsuhsc.edu

**Keywords:** SIV, alcohol, macaque, hippocampus, neural progenitor cells, Tat, microarray, neuroinflammation, neurogenesis

## Abstract

Alcohol use disorders (AUD) exacerbate neurocognitive dysfunction in Human Immunodeficiency Virus (HIV+) patients. We have shown that chronic binge alcohol (CBA) administration (13–14 g EtOH/kg/wk) prior to and during simian immunodeficiency virus (SIV) infection in rhesus macaques unmasks learning deficits in operant learning and memory tasks. The underlying mechanisms of neurocognitive alterations due to alcohol and SIV are not known. This exploratory study examined the CBA-induced differential expression of hippocampal genes in SIV-infected (CBA/SIV+; *n* = 2) macaques in contrast to those of sucrose administered, SIV-infected (SUC/SIV+; *n* = 2) macaques. Transcriptomes of hippocampal samples dissected from brains obtained at necropsy (16 months post-SIV inoculation) were analyzed to determine differentially expressed genes. MetaCore from Thomson Reuters revealed enrichment of genes involved in inflammation, immune responses, and neurodevelopment. Functional relevance of these alterations was examined in vitro by exposing murine neural progenitor cells (NPCs) to ethanol (EtOH) and HIV trans-activator of transcription (Tat) protein. EtOH impaired NPC differentiation as indicated by decreased βIII tubulin expression. These findings suggest a role for neuroinflammation and neurogenesis in CBA/SIV neuropathogenesis and warrant further investigation of their potential contribution to CBA-mediated neurobehavioral deficits.

## 1. Introduction

The lifetime history of alcohol use disorder (AUD) in persons living with Human Immunodeficiency Virus/Acquired Immune Deficiency Syndrome (HIV/AIDS) (PLWHA) is 55%, compared to 12%–29% in the general population [1,2]. AUD in PLWHA accelerates disease progression and contributes to comorbid pathologies, including cognitive dysfunction, termed HIV-associated neurocognitive disorder (HAND) [3,4,5]. This disorder occurs in up to 50% of PLWHA and is diagnosed when impairment is present in two or more cognitive domains (e.g., learning and memory, executive functioning, speed of information processing, motor skills, language) [6,7]. Joska et al. [3] demonstrated an interaction between alcohol use disorder and time from HIV diagnosis in the development of HAND. Moreover, history of alcohol dependence was associated with greater neurocognitive deficits in persons older than 60 [4].

Studies from our laboratory have demonstrated that chronic binge alcohol (CBA) administration in non-antiretroviral treated, simian immunodeficiency virus (SIV)-infected macaques increases viral load at set-point, increases muscle wasting, accelerates time to end-stage disease, and unmasks cognitive impairment as determined by an operant learning and memory task [8,9,10,11,12]. The operant learning and memory procedure required macaques to learn a sequence of lever presses daily and emit a previously learned sequence of lever presses to obtain food reinforcement. Our results found that SIV-infected animals receiving alcohol (CBA/SIV+) made more errors in these tasks than non-SIV-infected animals receiving alcohol (CBA/SIV−), SIV-infected animals (SIV+) and control animals (SIV−). Moreover, this was remarkable because these error-increasing effects were not evident on the days of the week when the CBA/SIV+ animals were not receiving alcohol. These types of operant tasks are associated with hippocampal function, an area of the brain shown to be negatively affected by both alcohol and HIV [13,14,15].

The underlying mechanisms by which CBA contributes to cognitive impairment in SIV-infected macaques are unknown. Studies utilizing an accelerated model of central nervous system (CNS) HIV disease in non-human primates indicate that inflammation correlates with CNS pathology [16,17,18]. Neuroinflammation also plays an important role in alcohol neuropathology [19]. We propose that the combination of chronic alcohol administration and HIV/SIV infection may exacerbate neuroinflammation and neuropathology. In order to explore potential mechanisms by which CBA may contribute to behavioral deficits, we performed microarray analysis of hippocampal tissue to determine differential patterns of gene expression in CBA/SIV+ macaques compared to SIV+ macaques. Based on the differential gene expression seen in the microarray, and the pathways enriched with a greater number of differentially expressed genes, we hypothesized that CBA impairs neural progenitor cell (NPC) differentiation.

NPCs are the source of new neuron and glial cells. These cells are found primarily in the subventricular zone and the hippocampus. We chose to further investigate the development process for several reasons: (1) the hippocampus is one of the primary sites of adult neurogenesis [20]; (2) alterations in adult neurogenesis have been linked to cognitive impairments [20,21]; (3) neuroinflammation alters neurogenesis [22,23,24]; and (4) experimentally impairing hippocampal neurogenesis results in learning and memory deficits [25,26,27]. Enhanced neuroinflammation, which is suggested by the microarray gene changes, alters neurogenesis. To further investigate one of the potential mechanisms of cognitive impairment suggested by the microarray analysis, we used an in vitro model to examine the impact of exposure to ethanol (EtOH) and HIV viral protein (Tat) on NPC differentiation. These in vitro studies allowed us to examine the functional relevance of the alterations in genes involved in neurogenesis. Our results provide insight into the principal relevant mechanisms involved in CBA and SIV neuropathogenesis.

## 2. Results

### 2.1. Hippocampus and Cerebrospinal Fluid Viral Load

Viral DNA, which reflects infected cells, was detectable in the brain tissue of one sucrose-administered SIV-infected animal (SUC/SIV+) and both CBA/SIV+ animals. Viral RNA, which indicates viral replication, was present in one CBA/SIV+ animal but none of the SUC/SIV+ animals. The cerebrospinal fluid (CSF) viral loads in both CBA/SIV+ animals were higher than the CSF viral loads from SUC/SIV+ animals (Table 1). Confirmation of SIV infection and peripheral viral data was previously published for these animals [10].

### 2.2. Process Networks Enriched with Differentially Expressed Genes

**Figure 1:**
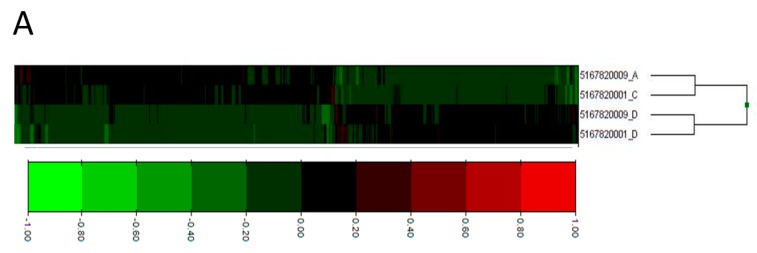

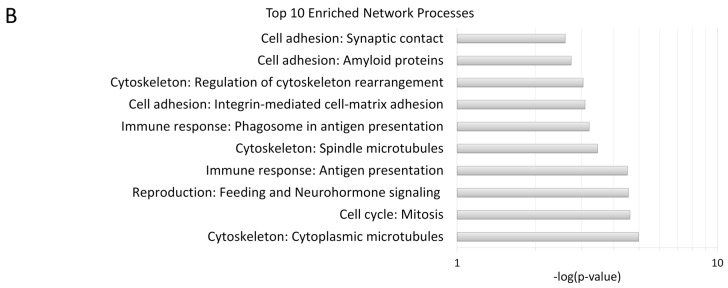
Heatmap of the differentially expressed genes in CBA/SIV+ and SUC/SIV+ used for MetaCore analysis and the top enriched process networks of those genes. (**A**) Heat map shows all 709 genes differentially expressed in CBA/SIV+ compared to SUC/SIV+. Differentially expressed genes were defined as the top one percent and bottom one percent of all genes detected in the microarray analysis. From those genes, only those in which both CBA/SIV+ animals had gene expression higher or lower than both SUC/SIV+ animals were used in process network analysis. Numbers 5167820009_D and 5167820001_D refer to SUC/SIV+ animals; (**B**) Top ten process networks enriched in differentially expressed genes in CBA/SIV compared to SUC/SIV macaques. Larger bars indicate greater significance.

**Figure 2:**
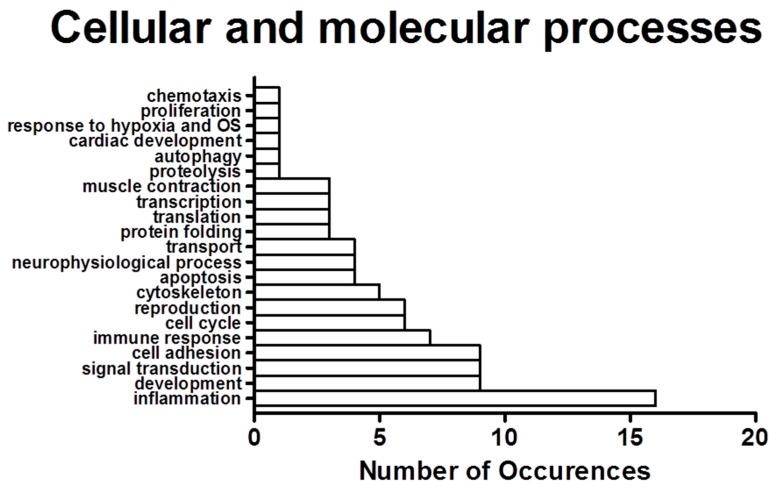
Number of occurrences in the top 100 specific process networks containing dysregulated CBA/SIV+ genes. The processes were classified using MetaCore nomenclature (e.g., the processes “inflammation complement system” and “inflammation IFN-gamma signaling” both count towards inflammation). The top 100 process networks containing differentially expressed genes were included in analysis. OS: oxidative stress; IFN: interferon.

**Figure 3:**
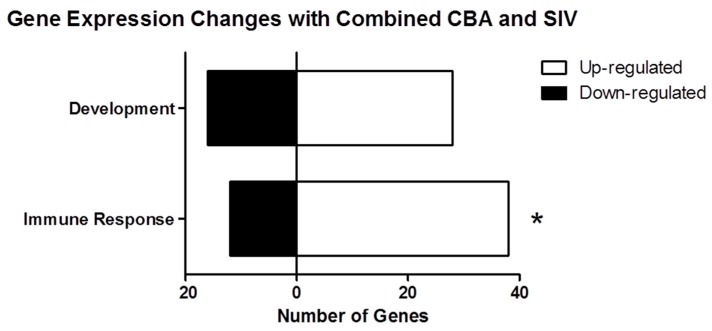
Number of genes involved in inflammatory and development processes that were up- and down-regulated. Immune processes include the set of genes from inflammation and immune response functions. The immune processes category had significantly more up-regulated genes than expected (76%) compared to the total number of up-regulated genes out of all differentially expressed genes (57%) (* *p* < 0.01, Chi-square test).

We determined the process networks of the differentially expressed genes using MetaCore from Thomson Reuters. These process networks identify categories and specific cellular and molecular functions of the differentially expressed genes. The top ten enriched network processes in hippocampi of CBA/SIV+ animals (Figure 1B) included several functions in the cytoskeleton, cell adhesion, and immune response categories. To complement the enrichment analysis, we also quantified the frequency of process categories (e.g., cytoskeleton, immune response) occurring in the top 100 processes in which the differentially expressed genes were classified. The total number of occurrences for each category (Figure 2) showed inflammation as the most numerous, with immune response processes also being relatively abundant. Notably, several processes within other categories were related to inflammation and immune response; specifically, “leukocyte chemotaxis”, “platelet aggregation” (cell adhesion) and “G1-S interleukin regulation” (cell cycle), indicating that the changes in immune function may contribute to changes in the other processes. To investigate this further, we combined “inflammation” and “immune response” gene lists into a single category, which we labeled “immune response”. We then determined the number of up- and down-regulated genes within this category (Figure 3). We found that there were significantly more up-regulated genes in this category than would be expected compared to the proportion of up-regulated genes detected in the microarray by chi-square test. The inflammation/immune response genes are shown in Table 2. Developmental, cell adhesion, and signal transduction process networks were the second most represented networks among all the top 100 processes.

Of the development process networks, three were involved in neurogenesis, specifically, “neurogenesis—axonal guidance”, “neurogenesis—synaptogenesis”, and “neurogenesis—general”, while five were involved in more general development processes, “Epithelial-mesenchymal Transition (EMT) regulation of epithelial-to-mesenchymal transition”, “hedgehog signaling”, “hemopoiesis, erythropoietin pathway”, “melanocyte development and pigmentation”, and “skeletal muscle development”. Twenty-eight of the neurogenesis genes were up-regulated and sixteen were down-regulated. Among the up-regulated genes were those involved in formation of new neurons and glial cells (e.g., *NEUROD1*, *OMG*, *PAFAH1B1*, *NHLH1*, *ITM2B*, *ZIC2* and *OLIG2*). However, the down-regulated genes also included genes involved in cell differentiation, axon guidance, and synapse formation (e.g., *PLXNA1*, *NRXN3*, *UNC5A*, *PSD95*, *NDP*, *NRG4* and *SOX8*). These changes in development genes led us to hypothesize that alcohol and SIV infection in combination could affect neurogenesis in the hippocampus, which could in turn explain the cognitive deficits seen in our previous studies [8]. To explore this possibility, we utilized an in vitro model to examine the combined effect of alcohol (EtOH) and the HIV protein Tat on NPC differentiation.

### 2.3. EtOH Treatment Alters NPC Differentiation in the Presence or Absence of HIV Tat

**Figure 4:**
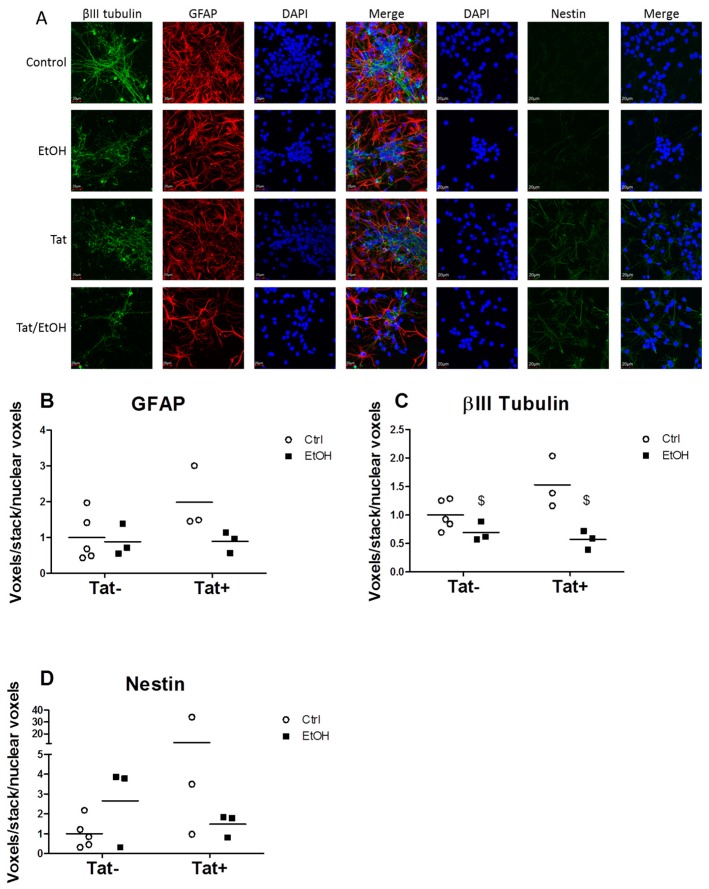
Panel (**A**) shows representative images from embryonic mouse neural progenitor cells (NPCs) treated with ethanol (EtOH) and/or trans-activator of transcription (Tat) on day one of proliferation and during four days of differentiation. Control staining is shown in the first row, EtOH in the second row, Tat in the third row, and Tat/EtOH in the fourth row. Quantification of immunofluorescence for each marker (βIII tubulin for neurons, glial fibrillary acidic protein (GFAP) for astrocytes, and nestin for neural progenitors) is shown in panels (**B**–**D**). Each symbol represents one experimental well. *N* = 5 for the control group and *n* = 3 for the experimental groups. Bar indicates the mean of each group. $ indicates main effect of alcohol (*p* < 0.05) by two-way ANOVA.

**Figure 5:**
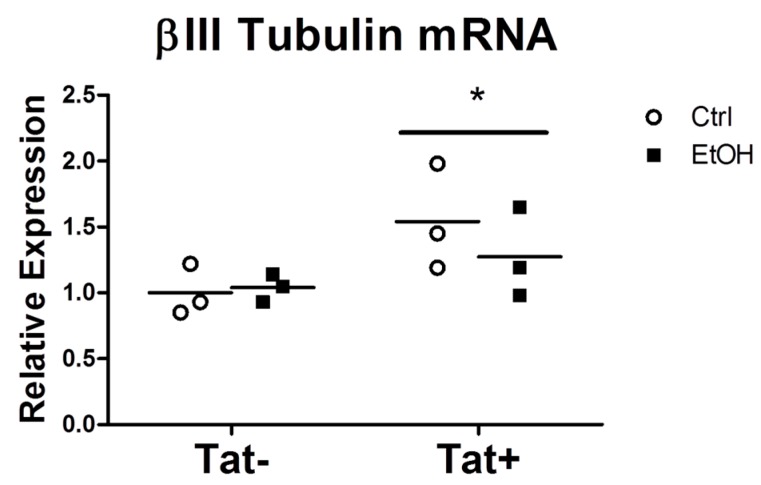
Relative βIII tubulin messenger ribonucleic acid (mRNA) expression in control (Ctrl) and EtOH treated NPCs with or without Tat. The solid line indicates the mean of each group. Data were analyzed by two-way ANOVA. *N* = 3 per group, * indicates *p* < 0.05 in Tat+ vs Tat-.

NPCs were cultured in the presence of EtOH, Tat, or combination of EtOH/Tat for five days, after which the cells were fixed and expression of neuronal, astrocytic, and NPC-specific proteins were determined by immunostaining. Representative images of NPCs immunostained for neurons, astrocytes, and neural progenitors are shown in Figure 4A. Two-way ANOVA analysis showed a significant main effect of EtOH (*p* < 0.05) in decreasing βIII tubulin staining (Figure 4C). There were no significant main effects or interactions in glial fibrillary acidic protein (GFAP) or nestin staining among any of the groups. Quantification of nuclear staining with 4',6-diamidino-2-phenylindole (DAPI) showed no differences among the groups, nor was there a difference in active caspase-3 staining. Together, these DAPI and caspase-3 data indicate that the decrease in βIII tubulin staining was not the result of changes in neuronal survival. We then quantified the gene expression of tubulin, beta 3 class III *(Tubb3)* to determine if EtOH inhibited βIII tubulin at the transcriptional level. Surprisingly, there was a small but significant main effect of Tat on *Tubb3* messenger ribonucleic acid (mRNA) expression, indicating that Tat affects the system pre-translationally and EtOH affects the system post-translationally (Figure 5A). The mRNA expression of several inflammatory genes detected in the SIV-infected macaque microarray data, along with several inflammatory cytokines implicated in HAND, were determined in NPCs. No significant differences in expression were seen in *H2-k1* (histocompatibility 2, K1, K region, murine equivalent of major histocompatibility class (MHC) I genes detected in microarray) or tumor necrosis factor receptor 1a (*Tnfrs1a*). Expression of pro-inflammatory cytokines *Tnf*, C-C motif chemokine ligand 2 (*Ccl2*), and interferon gamma (*Ifng*) was below the limit of detection for all groups.

## 3. Discussion

We examined the hippocampal gene expression profile of CBA/SIV+ macaques using microarray analysis and compared it with that of SUC/SIV+ macaques. The microarray results indicated there was differential expression of the genes involved in inflammation, immune response, development, cytoskeleton, and cell adhesion processes. We interpreted these results in the context of published findings indicating a relationship between increased expression of inflammation and immune response genes resulting in decreased neurogenesis, and impaired neurogenesis resulting in cognitive deficits [22,23,24,25,26,27]. From this framework, we then hypothesized that these changes in gene expression could indicate impaired neurogenesis resulting from the combination of alcohol and SIV. We then performed in vitro studies using isolated NPCs to test the hypothesis that EtOH and/or HIV Tat alter neurogenesis. These experiments revealed that EtOH treatment reduced expression of neuronal cytoskeletal protein βIII tubulin. These findings led us to speculate that neuroinflammation impairs neurogenesis and synaptic plasticity, which may be potential mechanisms by which CBA unmasks neurobehavioral deficits in SIV-infected macaques [8].

Up-regulation of both major histocompatibility class (MHC) I and II gene expression was observed in CBA/SIV+ macaques (e.g., human leukocyte antigen (*HLA)-F*, *HLA-A*, *HLA-E*, *HLA-DRA*). This CBA-specific up-regulation is potentially due to increased immunoproteasome activity. Supporting this interpretation, chronic alcohol administration increases the expression of immunoproteasome subunits and catalytic activity in brain tissue [28]. Furthermore, using this same model of CBA and SIV infection, we previously reported that proteasome subunit expression is dysregulated and proteasome activity is increased in skeletal muscle of CBA/SIV+ macaques [11]. Activation of the immunoproteasome favors MHC class I antigen presentation [29]. MHC proteins present peptides to immune cells as part of the innate and adaptive immune response. MHC protein expression is upregulated in microglia and macrophages of HIV-infected brains [30] and have been found to play a role in regulating synaptic density and function [31]. We speculate that our results indicating up-regulation of MHC gene expression could contribute to decreased synaptic density and function in hippocampi of CBA/SIV+ animals. However, models investigating the effect of MHCI manipulation have found variable outcomes on synaptic structure and function. MHCI knockout models have resulted in both reduced and increased neurite outgrowth, while overexpression has both enhanced and inhibited neurite outgrowth and synaptic density [32,33,34]. The cellular localization of MHCI receptors (neuronal vs immune cell) would likely affect the functional outcome of the change in MHCI expression. These relationships between immune response genes and synaptic function warrant further investigation.

We detected differential expression of genes involved in the growth and development of neurons, synaptogenesis, axonogenesis, and myelination in the hippocampus of CBA/SIV+ animals. Furthermore, these changes suggest that the combined effects of CBA and SIV infection hindered neurogenesis. The genes identified as having a role in synaptogenesis, such as post-synaptic density 95 (*DLG4*), contactin 1 (*CNTN1*), neurexin 1 (*NRXN1*), synaptotagmin 11 (*SYT11*), synaptotagmin 4 (*SYT4*), synaptotagmin 7 (*SYT7*), syntaxin 16 (*STX16*), actinin alpha 1 (*ACTN1*), synaptotagmin 14 (*SYT14*) and catenin beta 1 (*CTNNB*), are involved in synaptic plasticity [35]. These changes are important when considered in the context of the original goal of this exploratory study—to determine gene expression changes that could underlie cognitive impairment in CBA/SIV+ animals. Neurogenesis and synaptic plasticity in the hippocampus modulate learning and memory; impairment of neurogenesis or synaptic plasticity impairs learning and memory [20,21,36]. Neuroinflammation, a common feature of HIV infection or chronic heavy alcohol consumption [37,38], has been shown to inhibit neurogenesis [39] and synaptic plasticity [40]. Taken together, our findings, with previous reports in the literature, support our conclusion that the gene changes observed likely contribute to cognitive dysfunction in CBA/SIV+ macaques. Our results showing increased expression of immune function genes in CBA/SIV+ animals associated with alterations in nervous system development and synaptic plasticity genes are in agreement with published studies [22,24]. However, the importance of individual genes remains to be tested in vivo. The gene changes may not translate to cognitive impairments and further mechanistic experiments to determine the specific effects of the gene changes are warranted.

Our results and interpretation are based on gene expression changes in the absence of parallel cognitive measures in these animals, and can only be related to our previous findings using this model of CBA/SIV. However, microarray analysis of human brain tissue from HIV+ patients with neuropathology or cognitive impairments have revealed alterations in similar genes and cellular processes to those observed in our study. Masliah et al. [41] reported down-regulation of genes involved in transcription, ion channels/transporters, cell-cycle molecules, and neuronal cytoskeleton components along with increased expression of interferon-stimulated genes in frontal cortex isolated from HIV encephalitis (HIVE) brains compared to HIV+ brains. Moreover, Gelman et al. [42] compared the gene expression in the neostriatum, neocortex, and white matter of patients with HIVE and neurocognitive impairment (NCI) to that of HIV-patients. Those results indicate strong similarities between the changes in gene expression in HIVE brains and those reported here for brains of CBA/SIV+ animals based on increased expression of interferon signaling, antigen presentation, and complement system genes. Borjabad et al. [43] also showed that patients with HIV-associated neurocognitive disorders had enhanced expression of genes involved in antigen presentation and reduced expression of neurogenesis genes, giving further support to our results. The similarities in the processes found in human HIV brain tissue with neuropathology and/or cognitive impairment support our conclusion that alterations in immune processes/inflammation and neurogenesis in the CBA/SIV+ macaques could contribute to cognitive deficits.

The findings from the microarray suggesting the involvement of processes related to neuroinflammation and neurogenesis led us to explore the functional consequences of combined exposure to alcohol and HIV proteins in an in vitro model of neurogenesis. We used embryonic murine NPCs to model neurogenesis occurring in the adult hippocampus. This model allows for studying the early stages of neurogenesis in which NPCs commit to a mature astrocytes or neurons. Others have used a similar model to determine the effects of EtOH or Tat on neurosphere growth, as well as NPC proliferation and differentiation [44,45,46]. Our choice of 50 mM EtOH treatment matches what was used in previous neurogenesis studies [45] and matches the blood alcohol concentration achieved in the CBA/SIV+ macaques. Initially we aimed to validate gene expression of inflammatory receptors (*H2-ka*, *Tnfrs1a*) and pro-inflammatory cytokines (*Tnf*, *Ccl2*, *Ifng*) detected in the microarray in the NPC model. However, our results did not show any significant differences in their expression in response to EtOH and Tat exposure, which we believe is due to the absence of immune cells in this system, as the expression levels were low in all groups. In contrast, our results showed that Tat increased βIII tubulin at the mRNA level, while EtOH reduced the expression of βIII tubulin at the protein level. Together these results suggest that EtOH effects on βIII tubulin are post-translation. Future studies on this mechanism are warranted. The implications of this protein level change are relevant to cognition, as the expression of neuronal cytoskeleton proteins have been shown to be correlated to cognitive functioning in PLWHA. Moore et al. [47] showed that behavioral measures of cognitive functioning in humans correlated with the amount of immunohistochemical staining of the neuronal cytoskeletal protein microtubule-associated protein 2 (MAP2). The alteration of βIII tubulin expression with EtOH and Tat treatment indicates the functional relevance of the changes in neurogenic and synaptogenesis genes detected CBA/SIV+.

In summary, the results of this study show that CBA administration in SIV-infected macaques results in hippocampal up-regulation of genes involved in immune function and dysregulated expression of genes involved in neurogenesis. Similar patterns of gene expression are seen in brains of patients with HIVE and NCI [42], which we speculate suggests that over-activation of immune processes underlies the impairment of neurodevelopment. This is supported by the demonstrated capacity of inflammation to impair neurogenesis [23,39]. Thus, the results of this study support the hypothesis that inflammation-driven deficits in neurogenesis are a potential mechanism by which alcohol contributes to cognitive impairment in SIV-infected macaques. We recognize that involvement of several processes other than inflammation and neurogenesis were suggested by the microarray, including cytoskeleton related functions. These other processes may be important in contributing to cognitive deficits. There are limitations to our study, including the small number of animals in each group. However, this was an exploratory study with the goal of identifying potential mechanisms for further study, which we believe was accomplished. The timing at which gene changes were observed (19 months of CBA administration and 16 months of SIV infection) does not necessarily reflect time-dependent changes. Analysis at distinct time points would have provided valuable insight into the plasticity of the differential gene expression patterns observed in CBA/SIV+. It is important to note that the cell culture model used here does not contain all the cell types that would be found in vivo. The lack of microglia and infiltrating immune cells may account for the lack of inflammatory response in vitro. This was an exploratory study, and we believe that the Tat dose selected allowed us to investigate its effect on NPCs during growth and development. Others have used 200 ng/mL for neurogenesis experiments [46], and up to 1400 ng/mL to elicit inflammatory responses from astrocytes or produce neurotoxicity in culture [48,49]. The rich data set identifying genes and relevant biological processes should provide groundwork for future studies exploring time course, reversibility, and contribution of their changes to overall neurobehavioral dysfunction.

## 4. Materials and Methods

### 4.1. Animals

All experiments were approved by the Institutional Animal Care and Use Committee (codes 2522 and 3310) at both Tulane National Primate Research Center (TNPRC) in Covington, LA, and Louisiana State University Health Sciences Center in New Orleans, LA. All experiments adhered to the National Institutes of Health Guidelines for the Care and Use of Experimental Animals [50]. Animals had been used in previously published studies and the experimental procedures for alcohol administration and SIV inoculation are described in detail in those publications [10,51,52]. Briefly, young adult, male *Macaca mulatta* from two experimental groups; sucrose-administered SIV-infected (SUC/SIV+; *n* = 2), and CBA SIV-infected (CBA/SIV+; *n* = 2) were used in the study. SIV+ animals were all from the same experimental cohort and underwent all experimental procedures during the same time period. Animals were six years of age at necropsy.

CBA administration consisted of ethanol (30%) delivery via an indwelling gastric catheter providing a mean of 13–14 g/kg/week beginning three months prior to SIV inoculation and continuing throughout the study as previously described [10,51]. CBA administration was initiated prior to SIV infection to model risky alcohol use leading to HIV infection, as binge alcohol increases the risk of contracting HIV [53,54,55,56]. This protocol of alcohol administration results in blood alcohol concentrations ranging from 50 to 60 mmol/L, similar to that achieved with heavy alcohol consumption in humans [57]. The protocol of alcohol administration models a chronic binge-like alcohol intake, a frequent pattern of heavy alcohol consumption [58]. Following three months of CBA administration, animals were inoculated intravenously with 10,000 times the infective dose (ID_50_) of SIVmac251 (provided by Preston Marx, TNPRC). The progression of SIV disease was monitored throughout the study using clinical, biochemical, immunological, and plasma viral kinetic analysis as reported elsewhere [10,51,52]. Sixteen months post-SIV inoculation, animals were euthanized in accordance with the Panel on Euthanasia of the American Veterinary Medical Association. Whole brains were removed during necropsy, frozen in liquid nitrogen, and stored at −80 °C until analysis.

### 4.2. Hippocampus and RNA Isolation

Hippocampal brain tissue was isolated by dissecting tissue from a frozen hemi-brain. First, the intersection of the superior and inferior arcuate sulcus and the arcuate sulcus spur was located. Twenty millimeters caudal from that landmark, a coronal slice was made to reveal the hippocampus (bregma-20.00 mm) [59]. We then removed the hippocampus sample with a surgical scalpel.

RNA was extracted from frozen samples using TRIZOL reagent as recommended by the manufacturer (Invitrogen, Carlsbad, CA, USA). 200 ng of RNA were used to make biotinylated complementary RNA (cRNA) using the Illumina TotalPrep RNA Amplification Kit (Ambion, Austin, TX, USA), and hybridized for 14 h at 58° to HumanWG6_v3 chips, following manufacturer’s instructions (Illumina, San Diego, CA, USA). The arrays were scanned with the BeadArray Reader (Illumina, San Diego, CA, USA) and analyzed with GenomeStudio software (Illumina).

### 4.3. Cerebrospinal Fluid (CSF) Collection

CSF collection took place seven months after SIV inoculation. Macaques were anesthetized and a 22-guage needle was inserted into the fourth ventricle. CSF flowed freely from the needle hub and droplets collected until a volume of 0.5 to 1.0 mL was reached. CSF was stored at −80° C until use.

### 4.4. Viral Load Quantification

For quantification of viral loads, DNA and RNA were extracted from the frozen hippocampal tissue and CSF using DNeasy mini kit and RNeasy Universal mini kit following the manufacturer’s instructions (Qiagen, Valencia, CA, USA). SIV DNA and RNA levels were quantified with a TaqMan (Life Technologies, Carlsbad, CA, USA) quantitative PCR assay (qPCR) that targets a conserved region in SIV *gag* as detailed previously [60]. Briefly, the quantity of SIV DNA was measured in duplicate aliquots of DNA and normalized to cell number using a qPCR assay that targets a single copy gene (Rnase P). Copies of SIV RNA were determined by adding approximately 100 ng of sample RNA to duplicate reverse-transcription qPCR (rt-qPCR) amplification assays. The average SIV RNA copy number was determined and normalized to micrograms of RNA (for brain tissue) or mL CSF utilizing a rt-qPCR assay that targets the housekeeping gene ribrosomal protein S13 (*RPS13*), with validated Taq-Man primers and probe as described [61]. The limit of detection in these assays is 100 copies SIV DNA/1 × 10^6^ cells and 50 copies SIV RNA per microgram RNA.

### 4.5. Microarray Data Analysis

For Illumina chips, the background noise was eliminated by determining the level of hybridization of irrelevant probes. The signals were normalized assuming a similar distribution of transcript abundance in all the samples [62]. A differential analysis of gene expression was done using the level of expression in the SUC/SIV+ samples as a reference. A list of differentially expressed genes for CBA/SIV+ animals was generated by finding the top one percent of up-regulated genes and top one percent of down-regulated genes based on the frequency of fold-change values. This corresponded to the inclusion of genes that were increased or decreased 1.3 fold in CBA/SIV+ compared to SUC/SIV+, as used by other investigators [63,64]. The list of differentially expressed genes was further filtered by removing any genes for which there was overlap of their raw expression values between animals in the two groups. Thus, the final gene list contained the top one percent of up-regulated and top one percent of down-regulated genes in which both CBA/SIV+ animals had increased or decreased expression compared to both SUC/SIV+ animals (Figure 1A). In order to determine the biological significance and function of the differentially expressed genes, we used MetaCore from Thomson Reuters (Philadelphia, PA, USA) to determine the Process Networks of the differentially expressed genes [60]. The Process Networks indicate the cellular functions regulated by the input list of genes. We used two quantification strategies to analyze the MetaCore results in order to account for the multiple potential interpretations of these data. In the first analysis, we identified the most significantly enriched processes in which the differentially expressed genes are involved. In the second analysis, we quantified the top 100 processes of the differentially expressed genes. These analyses allowed us to determine the specific functions and general processes that are most affected by the combination of CBA and SIV infection. GeneCards (version 3.12.256, www.genecards.org, Rehovot, Israel) [65] database was used for determining the function of individual genes/proteins.

### 4.6. NPC Isolation and Culture

Using a standard technique [66], NPCs were isolated from pooled male and female mouse brains at embryonic day 17.5. After dissociation by gentle trypsinization (TrypleE Express, Gibco, Waltham, MA, USA) and pipetting with a fire-polished glass Pasteur pipette, single cell suspension neural cells were plated on non-adherent cell culture dishes at densities ranging from 1 × 10^2^ to 1 × 10^5^ cells/cm^2^. The culture medium used to support proliferation of neural progenitors and the formation of primary neurospheres consisted of NeuroBasal Medium (Gibco), B27 supplement (Gibco), N2 supplement (Gibco), Heparin (2 ng/mL), epidermal growth factor (EGF) (20 ng/mL, Invitrogen, Waltham, MA, USA), basic fibroblast growth factor (bFGF) (20 ng/mL, Invitrogen), and Glutamax (Gibco). Following three days of continuous growth, primary neurospheres were dissociated to single cell suspension and plated at low density (5.0 × 10^5^ cells/cm^2^) on non-adherent cell culture dishes in the same medium. The resulting secondary neurospheres were treated with or without EtOH (50 mM) and with or without Tat (200 ng/mL), for 24 h. The EtOH concentration was selected to match that achieved in vivo with the intragastric alcohol delivery protocol in the macaques. Tat concentrations were based on [46]. These non-adherent cells were then transferred to poly-D-lysine/Laminin coated glass chambers slides and allowed to differentiate in NeuroBasal medium containing B27 and N2 supplements, heparin, and Glutamax. During the differentiation process, the neurospheres were treated with EtOH (50 mM), Tat (200 ng/mL) or both on the first day of differentiation and EtOH (50 mM), Tat (125 ng/mL), or both on the third day of differentiation. The lower concentration of Tat on the third day was used to avoid overt cell death as we sought to determine how the treatments would alter cell development. Cells were fixed on day four of differentiation in the same chamber slides in which they were differentiated using 4% formaldehyde in phosphate buffered saline. Fixed cells were used for immunofluorescent analysis (*n* = 5 for controls or 3 for experimental groups); unfixed cells were pelleted and frozen at −80° C until use in Western Blot or qPCR assays (*n* = 3 per group).

### 4.7. NPC RNA Isolation and qPCR

NPC differentiation was determined by measuring the expression of the cytoskeletal protein nestin. The nestin expression decreases as the cells differentiate into neurons or astrocytes. New neurons express βIII tubulin and astrocytes express glial fibrillary acidic protein (GFAP). Evaluating the expression of each of these proteins allowed us to determine patterns of NPC differentiation into mature cell types. Measuring βIII tubulin and GFAP expression has been used by others to model the effects of HIV or alcohol alone on neurogenesis [44,45,67]. We utilized the RNeasy Mini Kit (Qiagen) to extract RNA from cell pellets. RNA purity and concentration was determined using spectrophotometry (NanoDrop, Wilmington, DE, USA). We reverse transcribed isolated RNA to complimentary DNA (cDNA) using QuantiTect Reverse Transcription Kit (Qiagen. *Tubb3* (NM_023279.2), *Gfap* (NM_010277.3), nestin (*Nes*) (NM_016701.3), *H2-ka* (NM_001001892.2), *Tnfrs1a* (NM_011609.4), *Tnf* (NM_013693.3), *Ccl2* (NM_011333.3), *Ifng* (NM_008337.4) and *Rps13* (NM_026533.3) primers were purchased from Qiagen . See Table 1 for RefSeq Accession numbers. The relative gene expression was quantified using the ∆∆Ct method with *Rps13* as the housekeeping gene.

### 4.8. NPC Immunocytochemistry

Slides were analyzed by immunofluorescent labeling to discriminate the content of neural cell types with anti-βIII tubulin (1:100, Biolegend, San Diego, CA, USA), anti-GFAP (1:100, Millipore, Billerica, MA, USA), and anti-nestin (1:100, Millipore, Billerica, MA, USA) antibodies diluted in 1% bovine serum albumin plus 0.02% Triton X-100. Secondary antibodies were goat anti-rabbit fluorescein- (Life Technologies, Carlsbad, CA, USA) or goat anti-mouse rodamine-conjugated (Pierce, Waltham, MA, USA) at 1:500 dilution in 5% bovine serum albumin plus 0.02% Triton X-100. Images were taken at 600x magnification using an Olympus Fluoview FV1000 Biological Laser Scanning Microscope equipped with a multi-line Argon laser (458 nm, 488 nm, 515 nm) and diode lasers (405 nm, 559 nm, 635 nm, Olympus of America, Center Valley, PA, USA). Imaging was done at 8 microseconds per pixel, 0.331 micrometers per pixel, and images were 640 × 640 pixels. Lasers were used at the following powers: 405 nm (20.0%), 488 nm (10.0%), and 559 nm (20.0%). Images were all captured using a 60x oil objective at 1x zoom. Software used was FV10-ASW version 4.00.03.04. Images (five to ten per slide) were all collected and quantified using the same settings. Z-stack images were taken from the top to the bottom of the cells within the view. Quantification of immunolabeling was accomplished using SlideBook 5.0 image analysis software version 5.0.0.19 (3i, Denver, CO, USA). MD performed the image acquisition and JM performed the image analysis. Identical thresholds for positive staining were applied to all images to determine the number of positively stained voxels. The number of positively stained voxels was normalized by both the thickness of the z-plane and by the number of nuclear voxels to account for variations in image thickness and cell number. Experimental groups are expressed as fold-change relative to control group.

### 4.9. Data Analysis

We used two-way ANOVA with Bonferroni post-hoc tests to analyze immunohistochemistry data obtained from the neural progenitor cell cultures. A chi-square test was used to compare the number of up- or down-regulated genes in process networks using the percentage of up- or down-regulated genes among all differentially expressed genes as the expected frequency. Statistical analysis was performed using GraphPad Prism (version 5.04 for Windows, GraphPad Software, La Jolla, CA, USA) and GraphPad QuickCalcs (web site: http://www.graphpad.com/quickcalcs/chisquared2/, accessed on 9 May 2016).

## Figures and Tables

**Table 1:** Simian immunodeficiency virus (SIV) DNA and RNA copy numbers in hippocampal tissue isolated at necropsy and cerebrospinal fluid (CSF) seven months prior to necropsy. Copy numbers of SIV gag DNA and RNA were normalized to the amount of housekeeping gene, ribosomal protein S13 (*RPS13*), present and then compared with a standard curve of known SIV copy number. CSF and plasma viral load were normalized to volume of CSF and plasma, respectively. CSF and plasma viral load were taken seven months prior to necropsy, during the asymptomatic stage of infection. The limit of detection was 25 copies in the tissue samples and 100 copies in the CSF. SUC/SIV+: sucrose administered, SIV-infected; CBA/SIV*+*: chronic binge alcohol administered, SIV-infected.

SIV DNA and RNA in Central Nervous System Compartments				
	Viral Load in Hippocampal Tissue		CSF Viral Load	Plasma Viral Load
Animal ID	DNA (SIV Copies/1 × 10^6^ Cells)	RNA (SIV Copies/μg *RPS13*)	RNA (SIV Copies/mL)	RNA (SIV Copies/mL, Log Transformed)
EK21 (SUC/SIV+)	<25	<25	<100	6.41
EJ59 (SUC/SIV+)	510	<25	5705	5.97
EN44 (CBA/SIV+)	140	<25	7560	5.59
EN72 (CBA/SIV+)	660	2267	112,000	5.79

**Table 2:** Inflammation and immune response genes that are differentially expressed between CBA/SIV and SUC/SIV macaques. FC is the fold-change in gene expression between CBA/SIV and SUC/SIV macaques.

Inflammation and Immune Response Associated Genes		
Gene	Description	FC
*TXNIP*	Mediator of oxidative stress, over-expression induces G0/G1 cell cycle arrest, required for maturation of NK cells	2.43
*CCL8*	Chemotactic factor for monocytes, lymphocytes, eosinophils, and basophils	1.91
*PROS1*	Anticoagulant plasma protein, stimulates fibrinolysis	1.82
*HLA-A*	Presentation of antigens to the immune system, major histocompatibility class (MHC) I	1.65
*C1QC*	Associates with C1r and C1s to yield the first component of the complement system	1.64
*CFB*	Complement Factor B, cleaved into Ba and Bb which further activate the complement cascade and monocyte response, Ba inhibits proliferation of pre-activated B-lymphocytes	1.60
*HLA-F*	Presentation of antigens to the immune system, MHC class I	1.58
*HLA-G*	Presentation of antigens to the immune system, MHC class I	1.57
*HLA-DRA*	MHC class II-type, expressed by B lymphocytes, dendritic cells, and macrophages; presentation of antigens to the immune system	1.54
*HLA-H*	Presentation of antigens to the immune system, MHC class I	1.52
*MSN*	Moesin, cross-linker between plasma membrane and actin cytoskeleton, localized to filopodia	1.52
*APP*	Functions in neurite outgrowth, neuronal adhesion, and axogenesis. Cleavage products of APP activate caspases and form amyloid plaques found in Alzheimer’s.	1.50
*TFRC*	Transferrin receptor, cellular uptake of iron, necessary for nervous system development	1.50
*ITPR1*	Intracellular inositol 1,4,5-triphosphate receptor, mediates release of Ca^2+^ from endoplasmic reticulum.	1.46
*MTOR*	Mediates cellular responses to DNA damage and nutrient deprivation	1.45
*JAK1*	Kinase in interferon-α, -β, and -γ signal transduction pathways	1.45
*EDG1*	Role in cell migration	1.45
*HLA-E*	Binds peptide-derived signal sequence of human leukocyte antigen (HLA) subtypes	1.45
*AP1G1*	Adaptin subunit, promotes formation of clathrin-coated vesicles for delivery from Golgi to lysosomes	1.44
*PIM1*	Proto-oncogene involved in cell survival, kinase activity suppresses pro-apoptotic proteins	1.44
*TNFRSF1A*	Tumor necrosis factor (TNF) receptor, activates nuclear factor kappa-light-chain-enhancer of activated B cells (NFkB), apoptosis, and inflammation	1.40
*ELK1*	Stimulates transcription induced by mitogen-activated protein kinase (MAPK) signaling cascade	1.39
*PSMA6*	Peptidase that is a component of the 20S subunit of the proteasome	1.39
*ODC1*	Important enzyme for polyamine synthesis, converts ornithine to putrescine	1.38
*NME1-NME2*	Naturally occurring read-through region between NME1-NME2 genes	1.37
*HLA-DPA1*	MHC class II-type, expressed by B lymphocytes, dendritic cells, and macrophages; presentation of antigens to the immune system	1.37
*GNG10*	G protein subunit gamma	1.35
*GNG2*	G protein subunit gamma	1.35
*VWF*	Maintains hemostasis, promotes adhesion of platelets to sites of vascular injury	1.34
*PSMD10*	Regulates 26S proteasome, p53-independent apoptosis and NFkB	1.34
*PPP2CB*	Serine/threonine phosphatase, implicated in negative control of cell growth and division	1.34
*PSMD6*	Subunit of 26S proteasome which co-localizes with DNA damage, degrades ubiquiniated proteins	1.33
*SEC61A1*	Crucial role in insertion of membrane and secretory proteins in to endoplasmic reticulum	1.33
*CEBPA*	Coordinates proliferation and differentiation of myeloid progenitors	1.32
*PIK3CB*	Catalytic sub-unit of Phosphoinositide 3-kinase (PI3K), activation of signaling cascades involved in cell growth, survival, and proliferation	1.32
*AP3B1*	Subunit of adaptor protein complex, target cargos into vesicles for delivery into neurites and nerve terminals	1.31
*HLA-DMA*	Prepares MHCII peptide binding site for antigen binding	1.30
*AVP*	Antidiuretic action in kidney, vasoconstriction	1.30
*MAPK11*	p38 MAPK, mediates activation of cellular response to pro-inflammatory cytokines	0.75
*ACTN1*	F-actin cross-linking protein, anchors actin to intracellular structures	0.75
*ELMO1*	Involved in cytoskeletal rearrangements needed of phagocytosis of apoptotic cells	0.74
*PSMD13*	Subunit of 19S regulator of the 26S proteasome.	0.74
*TRAP1*	TNF-receptor associated protein, maintains mitochondrial function	0.69
*LPL*	Lipoprotein lipase, hydrolysis of triglycerides in circulating chylomicrons and very low density lipoprotein (VLDL)	0.65
*CYCS*	Cytochrome C, election carrier in mitochondrial election transport chain, initiates apoptosis when released from mitochondria	0.64
*TIAM1*	Connects extracellular signals to cytoskeletal activities, activates Ras-related C3 botulinum toxin substrate 1 (RAC1), Cell division control protein 42 homolog (CDC42)	0.64
*ISG15*	Important for innate immune response against virus, inhibits HIV viral budding. Secreted interferon-stimulated gene 15 (ISG15) induces natural killer cell proliferation, neutrophil chemotaxis, induces interferon-gamma (IFNγ).	0.63
*CEBPB*	Transcription factor that regulates immune and inflammatory responses	0.59
*RYR1*	Ryanodine receptor, mediates release of calcium from intracellular stores	0.57
*HSPA1B*	Member of heat shock protein 70 family, stabilizes proteins against aggregation and mediates folding of new proteins	0.53
